# Multifunctional Conductive Paths Obtained by Laser Processing of Non-Conductive Carbon Nanotube/Polypropylene Composites

**DOI:** 10.3390/nano11030604

**Published:** 2021-02-28

**Authors:** Federico Cesano, Mohammed Jasim Uddin, Alessandro Damin, Domenica Scarano

**Affiliations:** 1 Department of Chemistry, University of Torino, Via P. Giuria, 7, 10125 Torino, Italy; alessandro.damin@unito.it (A.D.); domenica.scarano@unito.it (D.S.); 2 Photonics and Energy Research Laboratory, Department of Chemistry, The University of Texas Rio Grande Valley, Edinburg, TX 78539, USA; mohammed.uddin@utrgv.edu

**Keywords:** MWCNTs, polypropylene, MWCNT/PP composites, laser treatment, morphology, structure, electrical properties

## Abstract

Functional materials are promising candidates for application in structural health monitoring/self-healing composites, wearable systems (smart textiles), robotics, and next-generation electronics. Any improvement in these topics would be of great relevance to industry, environment, and global needs for energy sustainability. Taking into consideration all these aspects, low-cost fabrication of electrical functionalities on the outer surface of carbon-nanotube/polypropylene composites is presented in this paper. Electrical-responsive regions and conductive tracks, made of an accumulation layer of carbon nanotubes without the use of metals, have been obtained by the laser irradiation process, leading to confined polymer melting/vaporization with consequent local increase of the nanotube concentration over the electrical percolation threshold. Interestingly, by combining different investigation methods, including thermogravimetric analyses (TGA), X-ray diffraction (XRD) measurements, scanning electron and atomic force microscopies (SEM, AFM), and Raman spectroscopy, the electrical properties of multi-walled carbon nanotube/polypropylene (MWCNT/PP) composites have been elucidated to unfold their potentials under static and dynamic conditions. More interestingly, prototypes made of simple components and electronic circuits (resistor, touch-sensitive devices), where conventional components have been substituted by the carbon nanotube networks, are shown. The results contribute to enabling the direct integration of carbon conductive paths in conventional electronics and next-generation platforms for low-power electronics, sensors, and devices.

## 1. Introduction

Zero-dimensional (0D), 1D and 2D carbon structures have the potential to become materials of the next generation to be used in a series of applications, ranging from neuroscience [1] to energy fields [2]. As for materials for energy applications the research has been very active in recent years [3] and the interest includes, among other fields, supercapacitors [4], energy harvesting/storage [3,5], conversion devices [6,7], flexible electronics [8], sensors [9,10] and other electrochemical applications [11,12]. The limited amount in nature, the increasing price and demand for conventional materials (in particular metals) suggest that low-cost, high-performance and new materials would be desirable [13]. Due to their high conductivity, flexibility, mechanical properties and low weight, carbon nanotubes (CNTs) and graphene have been recently employed in the preparation of electrodes, fibers and polymer composites for electrical applications. 

As far as CNTs are concerned, they can be assembled into yarns [14] and then tested as wire conductors [13,15]. Such fibres, yarns or ropes, keeping the density very low, will most likely be the material for the next-generation conductors and they could have significant potential in the future development of electronic components [16], once control over the morphology, structure and doping allows outperforming any conventional metals for wiring (c.a. 10^4^ S cm^−1^ at room temperature) [13,17,18,19,20]. Notably, the best electrical performance is expected for high-purity, long, metallic SWCNTs [21]. Any increase in length would decrease contacts (i.e. junction resistance), while only single-walled carbon nanotubes (SWCNTs) and double-walled carbon nanotubes (DWCNTs) should be taken under consideration due to the fact that the two outermost walls of the nanotube seem to play positively in the electrical transport, while internal walls are adding weight only [21,22]. However, multi-walled carbon nanotubes (MWCNTs) are more suitable in practical applications for industrial-scale development owing to their lower production costs. 

In polymer composite materials, the electrical percolation is ruled by the filler type, shape and content [23,24]. In this regard, the formation of percolative paths at low filler loading with 1D/2D nanostructures may be preferred compared to spherically-shaped nanofillers or larger fillers [25]. There are some reports about the formation of segregated percolative paths inside the polymer composite [26,27,28,29,30], or conductive inks and coatings [31,32], but the spatial control of the electrical properties in these systems is still an open issue for practical applications. In this report, the application in the area of the laser stimulated percolation of CNT/polymer composites for low-power electronics is discussed. 

Although to date, the electrical properties of the obtained conductive tracks require further improvements, conductive paths made of pure carbon are enough to be employed as simple components, and in more complex circuits for the transport and control of low-current signals. For the first time in this study, it is shown that such conductive paths, easily obtained on more traditional MWCNT-based polymer composites, may be used as conventional components and that they can be easily connected with more traditional circuits. In this context, the present work is focused on the morphological/electrical property modifications and applications for simple electronic components, circuits and electrically sensitive devices made of carbon as a conductor. The laser printing method is in principle suitable for any thermoplastic and some thermosetting polymer composite materials containing carbon fillers (CB, CFs, CNTs, CNFs, graphite, graphene, GO, rGO) and it has been demonstrated to be effective, easy, and fast [8,33,34]. Other benefits include recyclability, low-cost, easy scalability, and possible integration into existing circuits [15,33,35,36]. 

Principles, basic results, and applications about the laser processing of graphite-, graphene- and MWCNT-based composites with the aim of increasing locally the electrical properties on the outer surface of non-conductive polymers are reported in [8,33,35,37,38,39,40]. In these works, a variety of polymers, including styrene-b-ethylene-co-butylene-b-styrene triblock copolymers (SEBS), PP, high-density polyethylene (HDPE), acrylonitrile–butadiene–styrene (ABS), polycarbonate (PC), ethylene–propylene–diene monomer (EPDM) rubber, epoxy resin, PP/PC and PC/ABS blends, and graphene oxide/polyethylene terephthalate (GO/PET) with MWCNTs were contemplated. However, some laser parameters, including the power at the sample surface (i.e., power density) and/or the accurate characterization of the transport properties are lacking. 

## 2. Materials and Methods

### 2.1. Material Preparation

MWCNT/polypropylene (PP) composites were melt compounded with the dilution of a masterbatch containing MWCNTs (NC7000 Nanocyl SA, Sambreville, Belgium) [41] and polypropylene-ethylene copolymer (Hostacom CR 1171 G, Lyondellbasell, Rotterdam, The Netherlands) filled with 12 wt.% of an inorganic filler and having a MFI = 11 g/10 min @230 °C. Composite specimens containing 1–4% by weight of MWCNTs, 3 mm thick injection-molded plates, were fabricated by injection molding technique by using a horizontal plastic injection moulding machine (Haitian MA900, Haitian Plastics Machinery Group Co., Ningbo, China) by using an injection time of 2 s, a speed of 30 cm^3^ s^−1^, at temperature of 230 °C under a pressure of 70 bar for 20 s, followed by cooling time of 18 s (total cycle time = 40 s).

The selection of the injection molding parameters comes from previous studies [42,43] highlighting the role played by the process parameters (i.e., injection rate and temperature, mould temperature) in affecting the electrical properties with pronounced anisotropy due to the lower conductivity of the external layers (i.e., skin effect), CNTs dispersion/aggregation and crystal polymer modifications during the heating and the cooling steps.

### 2.2. Laser Processing

**Figure 1:**
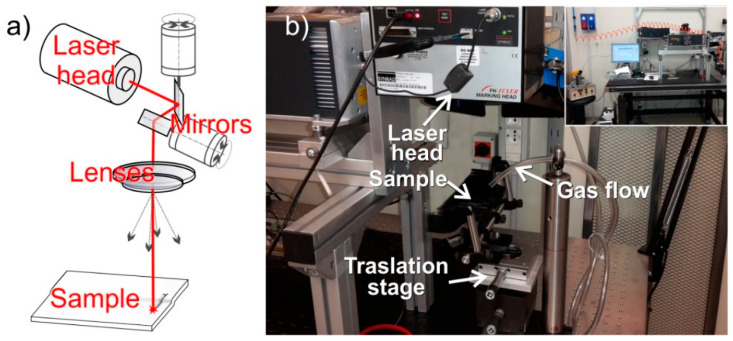
Schematic representation (**a**) and picture (**b**) of the laser printing equipment.

The laser (Firestar ti-series, Synrad, Mukilteo, Washington, USA) consists of a CO_2_ laser source (λ = 10.6 µm), equipped with a scanning head (FH Flyer, Synrad) with a focusing optics (200 mm focal length, FLA-200 optic lens, Synrad) operating in pulsed emissions (PWM type) with a maximum power of 60 W. By considering a beam quality (M^2^) > 1.2 and a Gaussian profile of the laser, a spot size of 310 µm (i.e. 1/e^2^) with a maximum power density of 1.59 × 10^5^ W/cm^2^ are obtained. This configuration allows the easy generation of simple geometries and more complex irradiation paths of different widths, lengths, and operations at different sample heights (1 cm) in a quite larger irradiated area (c.a. 15 × 15 cm). Laser parameters (frequency, power density and linear speed in the 5 ÷ 20 kHz, 4 ÷ 11 × 10^4^ 1,1 W/cm^2^, 10 ÷ 50 mm/s, respectively, were explored) combined with a repetition cycle in several operations, from the surface treatment to the cutting of specimens as obtained from the simple melting of the polymer to the ablation of the composite phases. The scheme and picture of the CO_2_-laser equipment are shown in Figure 1.

### 2.3. Material Characterization

Direct current (DC) electrical analyses of bulk regions and of conductive paths after laser irradiation were obtained by the two-probe resistivity measurements. The effect of the electrode contact was also verified by the four-probe method (four aligned and equally spaced electrodes).

Silver-based conductive paste was adopted to obtain ohmic contacts with a good low-resistance. Electrical properties were determined with a Keithley 2420 digital multimeter ( Keithley Instruments, Solon, USA).

The sample morphology has been investigated employing an Evo50 SEM instrument (Zeiss, Oberkochen, Germany) equipped with an energy-dispersive X-ray (EDX) detector (Oxford Instruments, Abingdon, UK). Before the morphological investigation, samples were cryo-fractured and chemically etched by KMnO_4_/H_3_PO_4_/H_2_SO_4_ solution for 15 h. The prototype top surfaces of the laser-irradiated regions were investigated without any chemical etching treatment. Both samples (cross-sections and top portions were covered by a 20 nm thick Au conductive film) and investigated at a medium acceleration potential (15 keV). 

The composition of MWCNTs and of the obtained MWCNT/PP composites were obtained by using different techniques, as follows: i) thermogravimetric analysis (TGA) by means of a TAQ600 (TA Instruments, New Castle, USA) analyzer with a heating rate of 10 °C/min up to 800 °C either in the air or under N_2_ gas flow followed by an isothermal step at 800 °C in air. The latter method was adopted to determine the polymer, carbon and inorganic contents after the compounding; ii) X-ray diffraction (XRD) analysis by utilizing a PW3050/60 X’Pert PRO MPD diffractometer (Malvern PANanalytical Inc., Malvern, UK), with a Cu anode and a Ni filter in Bragg–Brentano configuration, to identify the inorganic filler of the polymer on the TGA residue; iii) energy-dispersive X-ray spectroscopy (EDX) to identify inorganic impurities of MWCNTs. 

Raman spectra were acquired using an In Via Raman spectrophotometer (Renishaw plc, Wotton-under-Edge, UK), equipped with an Ar^+^ laser emitting at 514.5 nm. The laser beam was focused on the surface of samples through 20× ULWD objective. The effects of laser radiation over the samples were minimized by limiting the power to less than 1 mW at the sample surface.

Low-power DC prototypes were assembled with commercial components, including a 5 mm light-emitting diode (LED) HLMP-4700T-13/4 (Avago Technologies, San Jose, California, USA) with a forward voltage (V_f_) of 1.9 V at 2 mA, Arduino Uno (open-source board based on the ATmega328P microcontroller with 16 MHz clock) used for building electronics projects developed by Arduino.cc [44]. 

## 3. Results and Discussion 

**Figure 2:**
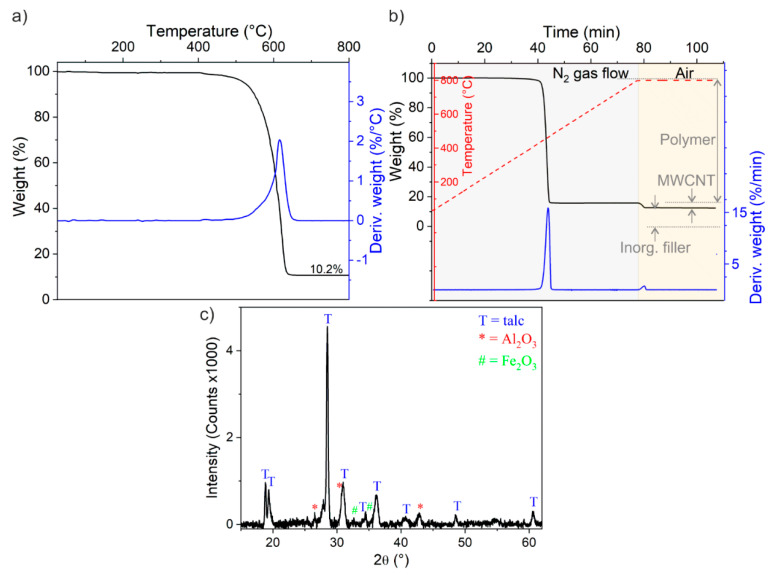
Thermogravimetric (TG) and difference thermogravimetric (DTG) plots (black and blue curves, respectively) of: (**a**) multi-walled carbon nanotubes (MWCNTs) in air, (**b**) melt-compounded MWCNT/polypropylene (PP) composite (4wt.% of MWCNTs) heated under N_2_ gas flow followed by an isotherm in air at 800 °C (the thermal profile is in red color); (**c**) X-ray diffraction (XRD) pattern obtained on residue after TGA thermal treatment. Talc (T peaks in the XRD pattern) is indexed with Magnesium silicate hydroxide (PDF card #29-1493) reference pattern. The XRD pattern could be entirely indexed with the reference patterns.

The composition of MWCNTs and of melt-compounded CNT-based polyethylene composites was obtained by means of EDX, TGA, and XRD measurements (Figure 2). The results are summarized in Table 1. It should be remarked that the Fe amount is around the EDX sensitivity limit and that geometry and density of the sample (i.e., bundled MWCNTs) are far from ideal conditions for EDX analysis. 

From Figure 2a and Table 1, it is clear that the MWCNTs do not consist entirely of carbon, but they contain some impurities that can be attributed to the catalyst support (Al_2_O_3_) and Fe nanoparticles used as a catalyst for the synthesis of MWCNTs. The total amount of impurities in MWCNTs was calculated to be about 10 wt.%. The Fe content resulted to be as low as c.a. 0.5 wt.%, as estimated by EDX analysis. These results are fully consistent with the literature about Nanocyl NC-7000 MWCNTs [41,45]. The same multi-technique approach was adopted to identify and quantify the contents of the polymer phase, inorganic fillers (i.e., talc) and of the MWCNTs in the melt-compounded samples. Briefly, the quantities of the polymer phase, inorganic filler and of MWCNTs in the composites were estimated from the weight losses (%) in the TGA plots during the heating step under N_2_ flow (quantity of polymer) and between the steps at 800 °C under N_2_ and air (quantity of MWCNTs) and residual weight % (quantity of inorganic filler) as shown for MWCNT/PP composites (3 wt.% of CNTs) in Figure 2b. Besides, talc was identified to be the exclusive inorganic filler in the polymer from the XRD pattern of the TGA residue of the composite. In conclusion, MWCNTs contain impurities whose major contribution is determined by Al_2_O_3_ with a minor presence of Fe nanoparticles. Furthermore, the amounts of MWCNTs in the melt-compounded samples were found to be as expected for composite compositions.

**Figure 3:**
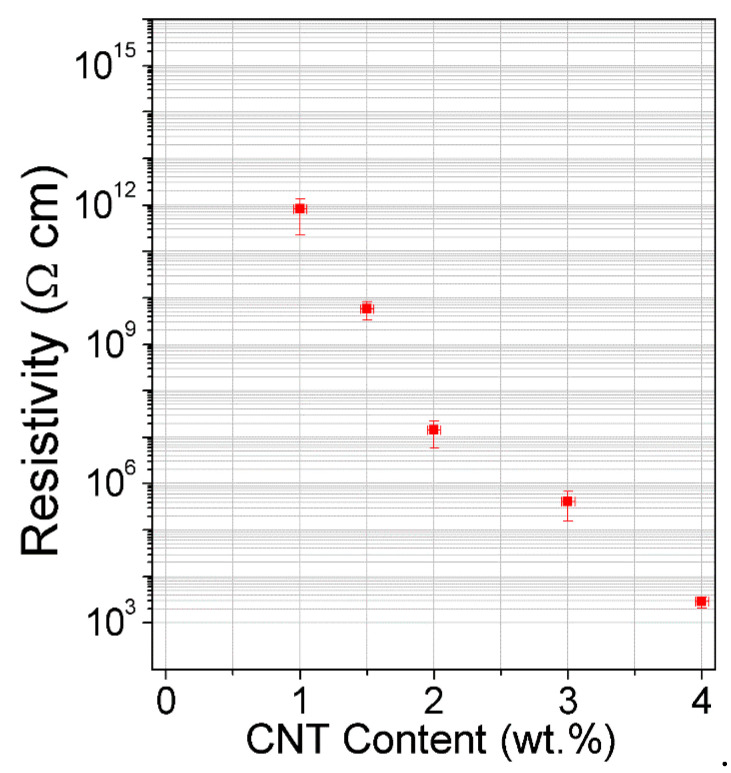
Electrical resistivity of MWCNT/PP as a function of the filler wt. percentage.

DC electrical properties of the melt-compounded MWCNT/PP samples, with 1–4 wt.% of MWCNTs, are shown in Figure 3.

**Figure 4:**
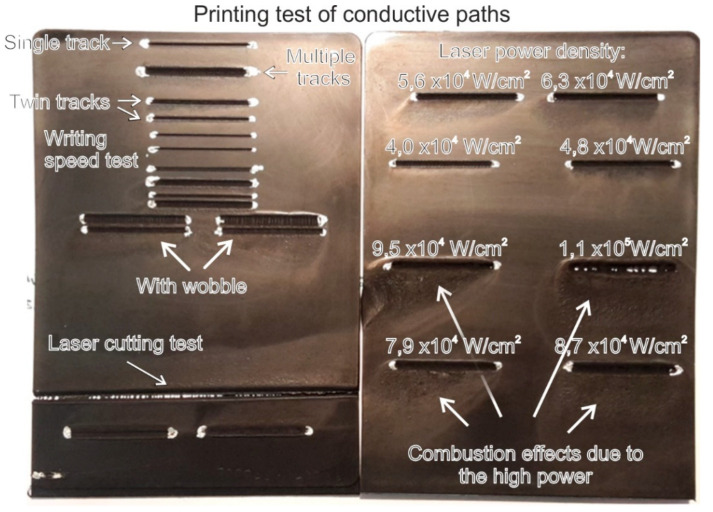
Effect of the laser processing parameters (power density, frequency, speed, repetition cycle, wobbler).

From this figure, it is clear that polymer composites with filler amounts as low as 1.5–2 wt.% fall near the electric percolation threshold, while undesirable effects are expected with higher filler amounts. A preliminary investigation of the laser interaction with 3 mm thick composite specimens has been performed although a precise analysis of the laser parameters has not been undertaken. Laser process parameters (power, frequency, speed, repetition cycle, defocusing) and flow conditions (N_2_, air) were tested on melt compounded samples containing 1, 1.5 and 2 wt.% of MWCNTs (Figure 4) in accordance with a previous study [33].

The purpose of this preliminary investigation was to nearly identify the effects produced by the laser by selecting suitable process parameters, due to the fact that the interaction of the laser with the CNT-based polymer composites may lead to many effects, including polymer ablation, welding and joining polymer sheets or cutting CNT fibers and films [33,34,46,47,48,49,50]. Among the various parameters considered, there are the power density, writing speed, frequency, and beam oscillation of laser. In Figure 4, an example of effects obtained with a single or a multitrack irradiation, as well as by varying the laser power and beam oscillation (laser wobbling) is shown. In our preliminary investigation the best results in the cutting test were obtained with 9 repetitions with a power density of 7.9 × 10^4^ W/cm^2^, f= 5 kHz, linear speed of 10 mm/s, while the best electrical properties were obtained with 20–40 repetitions, a power density of 2 × 10^4^ W/cm^2^, f = 15 kHz and with a linear speed of 200 mm/s, respectively. From these, single and a few parallel multiple irradiations were selected to spatially constrain the irradiated paths.

**Figure 5:**
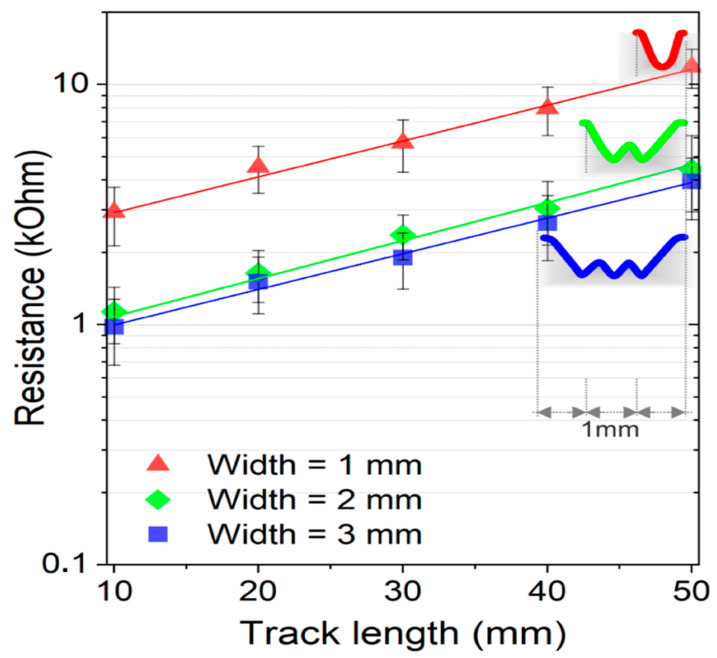
The electrical resistance of conductive tracks with different lateral sizes (1, 2 or 3 mm) and lengths (10, 20, 30, 40 and 50 mm) obtained on MWCNTs/PP polymer composite materials containing 1.5 wt.% of MWCNTs.

Composites containing 1.5 wt.% and 2 wt.% of MWCNTs were laser-treated to obtain conductive tracks 50 mm in length and 1, 2 or 3 mm in width, corresponding to a single irradiation path or to 1 mm spaced parallel irradiation paths. The 2 wt.% composition was excluded from the investigation, due to short-circuits of the order of tens of MΩ over an electrode distance of 1 cm between adjacent irradiated tracks. The electrical properties of the fabricated conductive paths are shown in Figure 5. 

In this plot it is shown that the resistance increases linearly with the track lengths (*l*) in the *l* = 10–20–30–40 or 50 mm range having a width of 1 and 2 mm. No consistent difference in resistance is observed for wider tracks (made by three parallel irradiation paths). We also verified by using the four-probe method [51] a non-negligible effect of the contact resistance (R_c_) of the electrodes. In this regard, the mean resistance of 50 mm conductive track decreased from 6.2 to 5.1 ± 1.2 kΩ when measured by a 4-probes linear configuration. 

**Figure 6:**
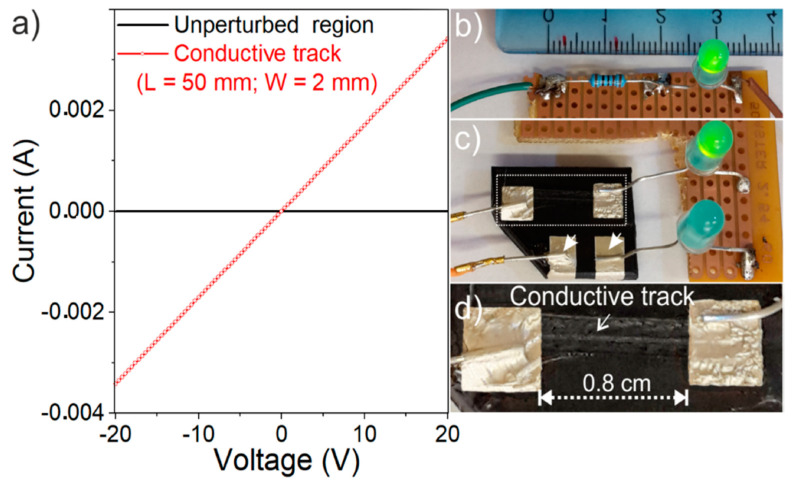
(**a**) V-I plot of MWCNT/PP polymer composite (1.5 wt.% of MWCNTs) and of a 50 mm long laser-irradiated region, as obtained by 4-probe measurements; a 3.6 V battery connected with: (**b**) a light-emitting diode (LED) with a commercial 1 kΩ series resistor, (**c**) a LED with a laser-stimulated conductive track of about 1 cm working as a series resistor (light on) or with the unperturbed region of the composite material (no brightness, here only for visual inspection and for comparison, everything was verified with the electrical investigation); (**d**) detail of the conductive track.

In Figure 6a the V-I characteristics of the laser-stimulated conductive track, obtained with the four-probe resistance measurements, are compared with the bulk properties of MWCNT/PP polymer composite (1.5 wt.% of MWCNTs). Interestingly, the measured V-I linear characteristics in the ±20 V interval suggest that conductive tracks can be used as resistors, whose resistance can be varied with the length and the width of the track. In practical use, the current passing through a light-emitting diode (LED) is usually limited by a small resistance, because such a diode has a very low heat transfer constant. In this regard, a portion of the conductive track was tested as a series LED resistor connected to a battery of 3.6 V in a DC low power electronics (Figure 6b–d), as schematized in Figure S1 (Supplementary File 1 and 2).

No significant variations were found after several working cycles. Notably, the same light brightness was obtained for the circuit prepared with a commercial resistor of 1 kΩ (±1%) (Figure 6b). As expected, no light was detected for electrical connection outside the laser-perturbed region of the composite (Figure 6c), as obtained without any resistance used as a second comparison. Although in the described experiment the joule effect exists (i.e., I × V), it does not need to be taken into account for the resistor due to the very low current. In a separated experiment, a 50 mm long laser stimulated conductive track connected with a potential of +20 V no significant variations of the electrical properties (power, current and resistance) with the time have been evidenced (Figure S2, Supplementary File 1). This experiment indicates that MWCNT-based conductive tracks work exactly as expected across the whole range (±20 V) and that no difference was observed when compared with commercial resistors. 

**Figure 7:**
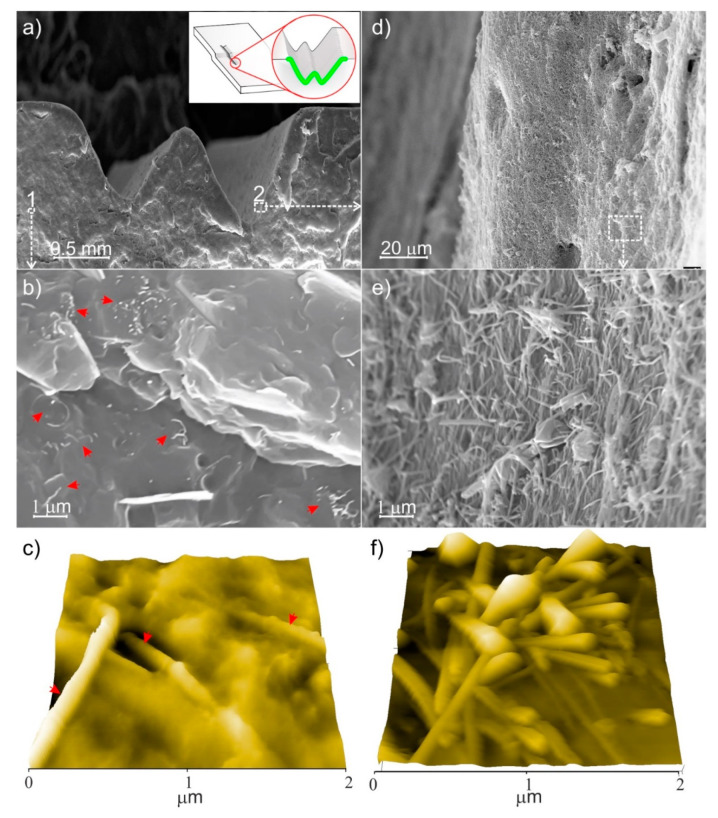
(**a**) Cross-sectional SEM image of the MWCNT/polymer composite (MWCNTs 1.5 wt.%) along the CO_2_-laser irradiated region; (**b**) SEM enlarged view of 1-selected area in (**a**) panel and (**c**) 3D AFM image, both representative of (untreated) and bulk regions; (**d**) and (**e**) SEM enlarged views collected at the different magnification of 2-selected area in (**a**) panel, (**f**) 2 × 2 µm 3D AFM image at the surface of the channel walls. Arrows in (**b**,**c**) correspond to isolated nanotubes/small bundles dispersed in the polymer matrix. In the inset of (**a**) the profile after the CO_2_-laser treatment obtained with a pair of two parallel irradiation paths.

SEM and AFM images of the conductive track of the MWCNT/polymer composite (MWCNTs 1.5 wt.%) are shown in Figure 7. These images can be considered representative of all tracks with good electrical characteristics. 

In these images, a cross-section of two regularly spaced grooves corresponding to a pair of two parallel laser irradiations forming a single conductive path, 2 mm wide and 500–750 µm depth, is illustrated (Figure 7a). The presence of individual MWCNTs or small CNT bundles (and of other inorganic fillers, such as talc particles) dispersed in the polymer matrix can be highlighted far from the irradiated regions (Figure 7b,c). By contrast, at the surface of the V-shaped profile corresponding to the laser irradiation path a continuous layer consisting of entangled MWCNTs is SEM and AFM imaged (Figure 7d–f). From these images, we can conclude the remarkable role of the laser irradiation treatment in the formation of an accumulation layer of MWCNTs, which are randomly distributed at the surface. From the SEM and AFM images, it is clear that the MWCNT accumulation layer corresponds to the electrically conductive paths. 

**Figure 8:**
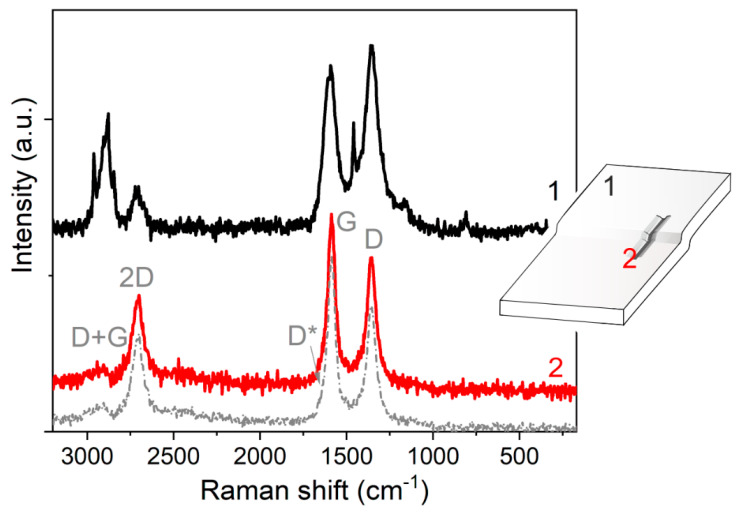
Raman spectra of: MWCNT/PP composite (black line, 1), CO_2_-laser irradiated regions (red line, 2) and of the pristine MWCNTs.

Raman spectra collected far from CO_2_-laser treated region (unperturbed region) and on CO_2_-laser treated region for the MWCNT-based PP composite (1.5 wt.%) are illustrated in Figure 8. 

These spectra can give information on the effects of the perturbation due to the laser irradiation as compared to the unprocessed composite material, and to the bare MWCNTs. A more detailed assignment of what is commented below is provided in Figure S3 (Supplementary File 1).

The Raman spectrum, obtained after laser beam irradiation (2-red spectrum), shows bands with maxima at c.a. 1356, 1586, 2703, and 2943 cm^−1^. Such fingerprints are called D, G, 2D (or G’), and D + G (or 2D’) bands [52], respectively. These bands can be assigned to the presence of defects in MWCNTs (D-band), in-plane vibration of the C–C bonds (G-band), and to the overtones of the D-band [53,54]. The shape and positions of all these fingerprints are undoubtedly similar to those of the bare MWCNTs. It is worth mentioning that due to the disorder, G and D′ peaks are broadened, and it is more convenient to consider them as a single G line. The lack of three-dimensional order in these materials is also confirmed by the disappearance of the doublet in the second order D peak [52]. Furthermore, the profile and the D- and G-peak shifting towards higher wavenumbers of the original MWCNTs and of the conductive tracks with respect to the composite material are compatible with the smaller intertube interactions in the polymer composite [55].

Raman spectrum of conductive tracks (2-red spectrum) show well separated and defined D- and G-bands with I_D_/I_G_ = 0,75, which is close to the intensity ratio of the bare MWCNTs (0,7). By contrast, the Raman spectrum of the untreated surface (1-black spectrum) shows a more complex profile. In addition to D-, G-, and 2D-bands, other features with maxima at 2961, 2906, 2883, and 2845 cm^−1^, then at 1459, 1167, and at 812 cm^−1^ are present. The band envelope in the range 2960–2840 cm^−1^ is due to symmetrical and asymmetrical C–H stretching [56], while signals at 1459 and 1167 cm^−1^ are associated with δ(CH_2_) and δ(CH), respectively [57]. Finally, the band at 812 cm^−1^ can be assigned to ρ(CH_2_), υ(C–C), and υ(C–CH) [58,59]. 

Interestingly, in the spectra obtained on the laser-irradiated region the polymer phase is not detected, and Raman fingerprints indicate the presence of an envelope of MWCNTs, while far from these regions, the classical PP bands in the 2960–2800 cm^−1^ and 1500–1000 cm^−1^ ranges are present together with weaker signals due to MWCNTs dispersed in the polymer. In conclusion, we can assume that an increase in the local concentration of MWCNTs in the laser-irradiated regions is obtained, but the laser-induced perturbation of MWCNTs cannot be ruled out.

**Figure 9:**
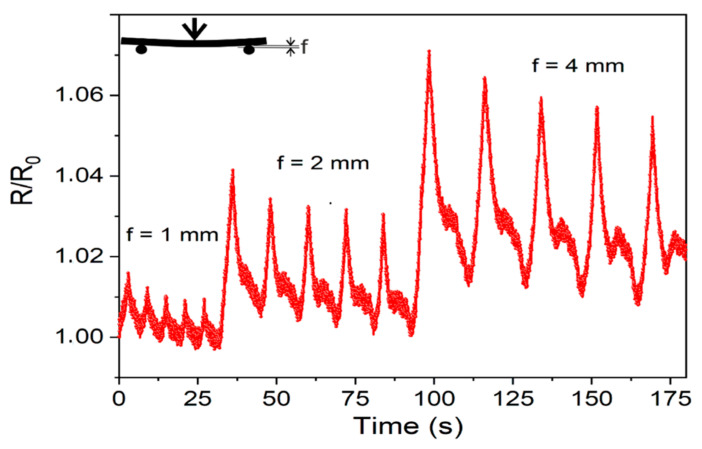
Piezoresistive properties of the laser-stimulated conductive tracks of 50 mm for repeated flexure cycles of 1, 2 and 4 mm.

The three-point flexural test of the conductive track is shown in Figure 9. 

The electric resistance (R/R_0_) was monitored under dynamic conditions during repeated deflection cycles of 1 mm, 2 mm and 4 mm. From this plot, it is clear that upon repeated flexural cycles, R/R_0_ is slightly reduced at lower values, but it rises sharply for increased flexures. The detailed explanation of the piezoresistive characteristics of the conductive paths is beyond the scopes of this paper, but it is worthy of attention the fact that the R/R_0_ drift can be observed for the same repeated flexures and that as bending increases a rebound piezoresistive peak, which is higher at increased flexure values, is observed during the deflection steps. Both these observations, together with the non-specular shape of the electric signal in the bending/unbending cycles can be attributed to the MWCNT rearrangements due to the viscoelastic nature [60]. Furthermore, a linear piezoresistive signal is usually observed under very low strains (ε ≤ 1%), while a non-linear piezoresistivity is obtained at larger deformations [61]. In other words, permanent modifications of the CNT network structure, due to the small van der Waals interactions acting in the bundled structure of the MWCNTs, are most likely achieved even at low deformations.

**Figure 10:**
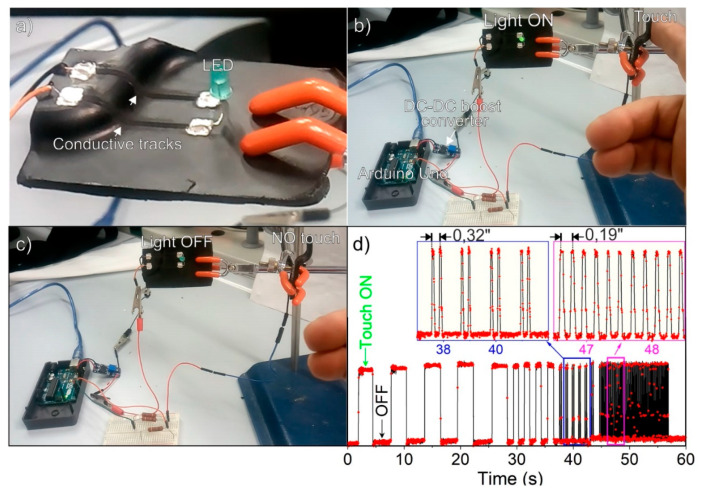
Proof of concept of the capacitive sensor connected with a LED and with conductive tracks: (**a**) two 50 mm long laser-stimulated conductive tracks connected with a light-emitting diode (LED), (**b**) brightness with the finger touching, (**c**) no brightness without touching, (**d**) time responsivity under ON/OFF touching cycles. Single and dual-touch tests are illustrated in the insets of (**d**).

Owing to some advantages, including low production cost and power consumption, nanocarbon polymer-based touch sensors connected with traditional electronics, have been recently proposed [62,63]. In this domain, the laser-stimulated conductive tracks were tested in a prototype circuit for transport and control of electrical signals, considering the good electrical characteristics for low-power electronics as LED series resistors (Figure 10a). The brightness of a LED was regulated by the laser stimulated conductive tracks, working as resistors, connected to I/0 pins of the Arduino Uno board, and controlled by the finger touching (Figure 10b,c) and as schematized in Figure S4 (Supplementary File 1). Although the voltage drop provided by the I/0 pins of the Arduino board was limited (5 V) and the resistance of the conductive tracks (c.a. 4,5 kΩ/track) was moderately high, a DC–DC boost converter module [64] was arranged between the Arduino I/0 pins and the LED prototype circuit for raising the voltage up to 20 V_DC_. The time responsivity under ON/OFF touching cycles in single and dual-touching tests are illustrated in Figure 10d, Figure S5 (Supplementary File 1), and Supplementary File 3 (video).

The results above illustrate that the CO_2_-laser irradiation can locally increase the conductivity on the surface of MWCNTs/PP composites, whose conductive filler content in the bulk is below the percolation threshold. The effect of the laser in increasing electrical conductivity by several orders of magnitude is associated with the local increment of the MWCNT concentration and the resulting resistance per length of conductive path is as low as about 1 kΩ/cm. To the best of our knowledge this value is among the best that has been achieved by the laser treatment considering the mixture of MWCNTs with other polymer matrices, including SEBS, PP, HDPE, ABS, PC, EPDM, epoxy resin, PP/PC and PC/ABS blends, and GO/PET [8,33,35,37,38,39,40]. Although the use of these technologies applied to conventional and unconventional carbon-based polymer composites may enable new applications and functionalities, the resistance values are still too high and represent a limitation for most applications due to the random distribution of CNTs as obtained by laser activation processes.

## 4. Conclusions

Carbon nanotubes are already employed as fillers in polymer composites for mechanical and electrical applications. However, developing a proper strategy to selectively obtain percolation paths in materials and understanding the mechanisms involved in the formation and the control of the properties, are key issues enabling next-generation smart materials. Interestingly, laser processing is a fast, simple and convenient method for the fabrication of electrically conductive paths in pre-existing insulating matrices, which contain a small amount of conductive fillers (i.e., with filler content far below the electrical percolation threshold). More interestingly, electrically responsive paths and embedded sensors based on MWCNT/polymer composites can be obtained and integrated with existing electrical circuits. Interestingly, by combining different investigation methods, including thermogravimetric analysis, X-ray diffraction measurements, scanning electron and atomic force microscopies, and Raman spectroscopy, the electrical properties of MWCNT/PP composites have been elucidated to unfold their potentials under static and dynamic conditions. The presented results show, for the first time, that laser-stimulated conductive tracks made of a carbon nanotube network work exactly as a resistor, whose resistance can be easily controlled by the laser process parameters. More interestingly, prototypes made of simple components and electronic circuits (resistor, touch-sensitive device), where conventional components have been substituted by the carbon nanotube networks, are shown. The results contribute to enabling the direct integration of carbon conductive paths in conventional electronics and in next-generation platforms for low-power electronics, sensors, and devices.

## Figures and Tables

**Table 1:** Composition of MWCNTs and of the compounded MWCNT/PP composites.

	Composition (wt.%)						
	C ^1^	O ^1^	Al ^1^	Fe ^1^	Polymer ^2^	Inorganic fillers ^2,3^	MWCNTs ^2^
MWCNTs	92	3.5	4	(0.5)		(talc)	
							
Pure polymer					88	12	-
1-MWCNT/PP					87.1	11.9	1
1.5-MWCNT/PP					86.7	11.8	1.5
2-MWCNT/PP					86.2	11.8	2
3-MWCNT/PP					85.4	11.6	3
4-MWCNT/PP					84.5	11.5	4

^1^ obtained from energy-dispersive X-ray (EDX) analysis. ^2^ calculated from TGA profiles. ^3^ phase identification from XRD patterns of the TGA residues.

## Supplementary Materials

The following are available online at https://www.mdpi.com/2079-4991/11/3/604/s1, Supplementary File 1. Figure S1: Scheme of the laser-stimulated conductive track working as a LED series resistor, Figure S2: Time dependence of electrical properties (power, resistance and current) of a 50 mm long the laser-stimulated conductive track connected with a potential of 20 V; Figure S3. Deconvolution and assignation of the Raman spectra fingerprints for PP and for MWCNTs far from (black spectrum, a), and in the CO_2_-laser irradiated (red spectrum, b) regions of the composite.; Figure S4: Layout of the fabricated laser-stimulated conductive tracks integrated with the Arduino board, Figure S5: a) touch sensing output from the capacitive sensing device under ON/OFF touching cycles of different duration and time responsivity in dual- and single touch tests. Supplementary File 2: A laser-stimulated conductive path working as a resistor, Supplementary File 3: Laser-stimulated conductive paths working as resistors in a touch sensor prototype connected with Arduino Uno board.

## Abbreviations

ABS: acrylonitrile–butadiene–styrene; AFM: atomic force microscopy; CB: carbon black; CFs: carbon fibers; CNFs: carbon nanofibers; CNTs: carbon nanotubes; DTG curve: first derivative of the TGA curve; DWCNTs: double-walled carbon nanotubes; EDX: energy dispersive X-ray; DC: direct current; EPDM: ethylene–propylene–diene monomer; GO: graphene oxide; HDPE: high-density polyethylene; LED: light emitting diode; MFI: melt flow index (of a polimer); MWCNTs: multi-walled carbon nanotubes; PDF: powder diffraction file; PET: polyethylene terephthalate; PP: polypropylene; PC: polycarbonate; PWM: pulse width modulation (of laser emission); SEBS: Styrene-b-ethylene-co-butylene-b-styrene triblock copolymers; R_c_: contact resistance; rGO: reduced graphene oxide; SEM: scanning electron microscopy; SWCNTs: single-walled carbon nanotubes; TGA: thermogravimetric analysis; ULWD: Ultra Long Working Distance; V_f_: forward voltage; XRD: X-ray diffraction; δ(CH_2_): CH_2_ bending vibrations; ρ(CH_2_): CH_2_ rocking vibrations; υ(CH): CH stretching vibrations.
