# Supplementary material for: Multifunctional Conductive Paths Obtained by Laser Processing of Non-Conductive Carbon Nanotube/Polypropylene Composites
Source: Nanomaterials (Basel). 2021 Feb 28;11(3):604. doi: 10.3390/nano11030604 (PMC7997224; doi:10.3390/nano11030604)
Supplement: Supplementary file 1 [file nanomaterials-11-00604-s001.zip › nanomaterials-1099304-SI/Supplementary File 1.pdf]

Multifunctional Conductive Paths Obtained by Laser Processing of Non-Conductive Carbon Nanotube/Polypropylene Composites

Federico Cesano [1,*], Mohammed Jasim Uddin [2], Alessandro Damin [1] and Domenica Scarano [1]

[1] Department of Chemistry, University of Torino, Via P. Giuria, 7, 10125 Torino, Italy; alessandro.damin@unito.it (A.D.); domenica.scarano@unito.it (D.S.)
[2] Photonics and Energy Research Laboratory, Department of Chemistry, The University of Texas Rio Grande Valley, 78539 Edinburg, TX, United States. mohammed.uddin@utrgv.edu (MJU)
* Correspondence: federico.cesano@unito.it; Tel: +39-011-6707548 (F.C.)

Supplementary Materials

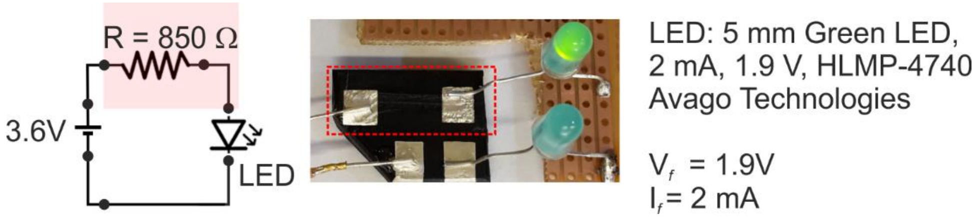

Figure S1. Scheme of the laser stimulated conductive track working as a LED series resistor.

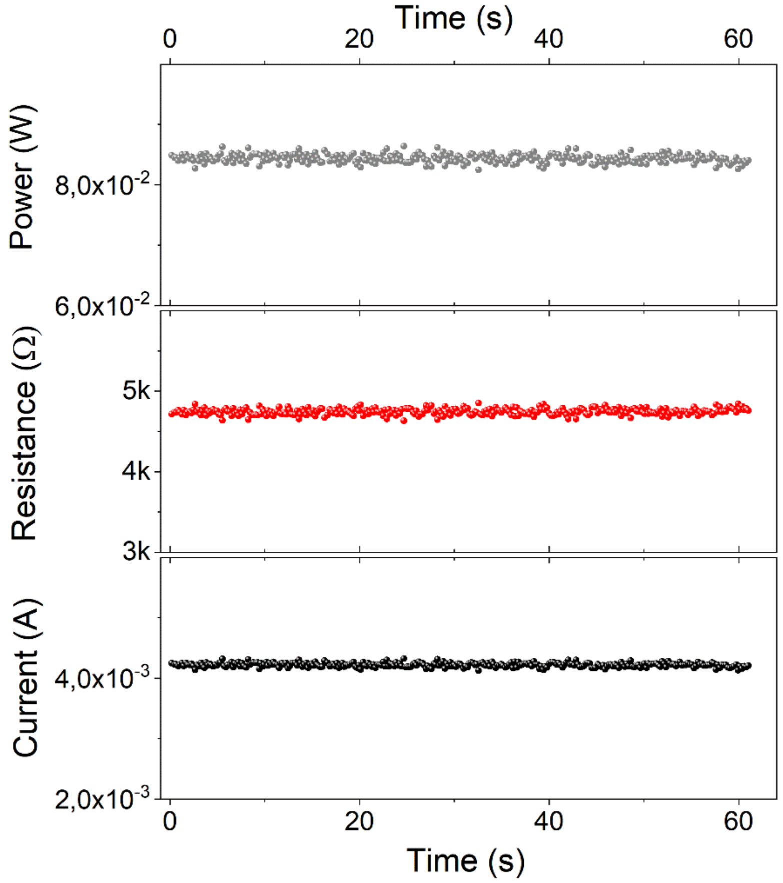

Figure S2. Time dependence of electrical properties (power, resistance and current) of a 50 mm long the laser stimulated conductive track connected with a potential of +20 V.

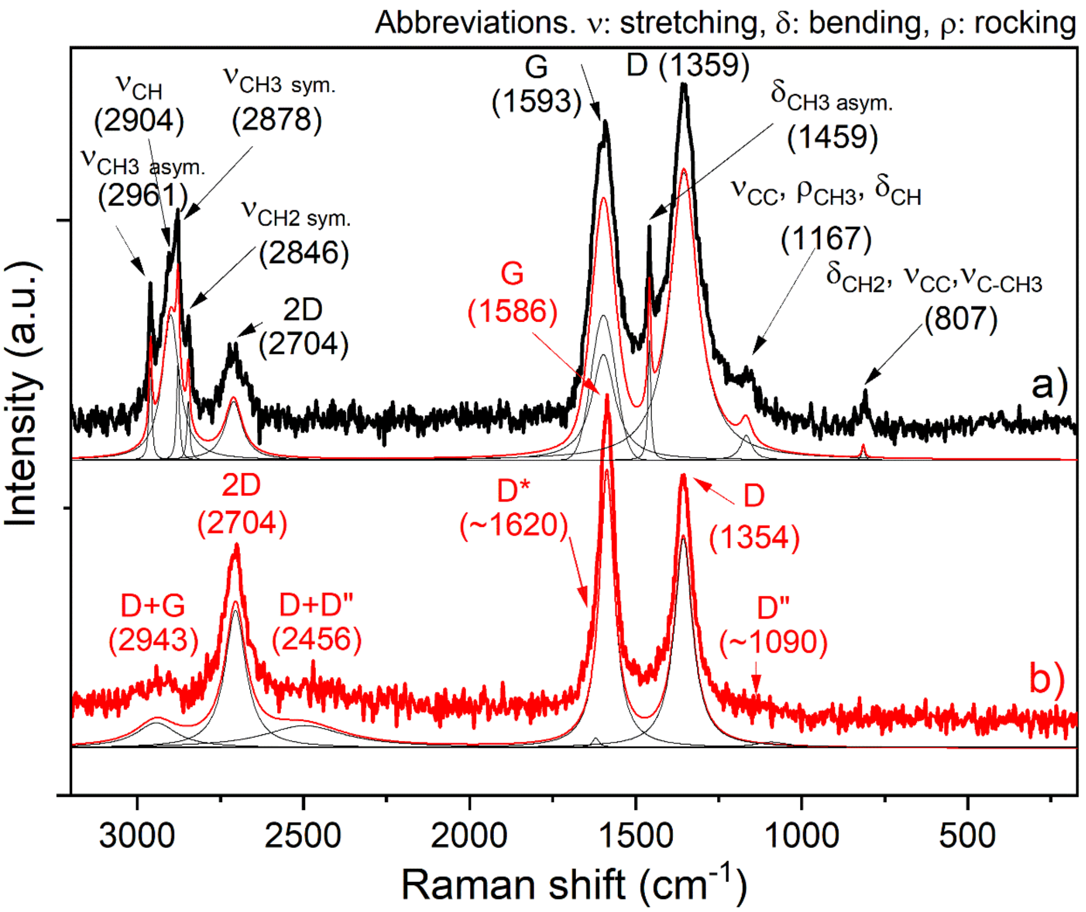

Figure S3. Deconvolution and assignation of the Raman spectra fingerprints for PP [1-3] and for MWCNTs [4-6] far from (black spectrum, a), and in the CO_2-laser irradiated (red spectrum, b) regions of the composite.

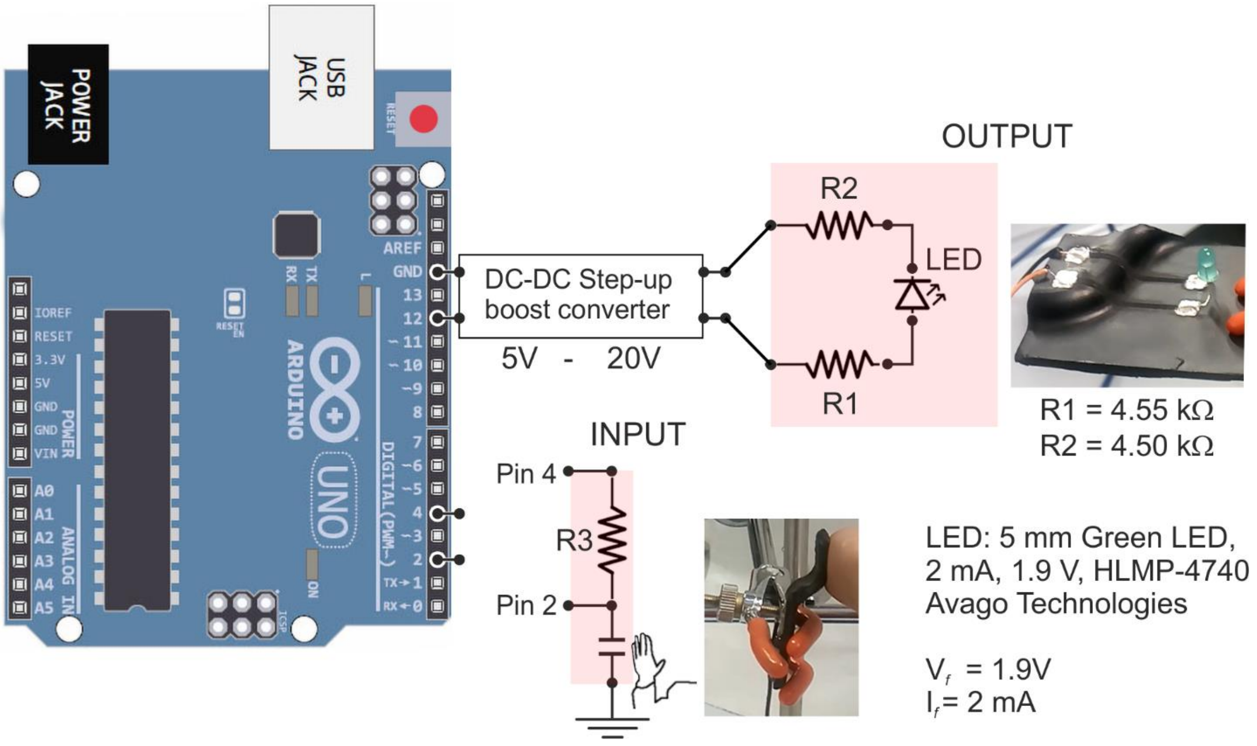

Figure S4. Layout of the fabricated laser-stimulated conductive tracks integrated with the Arduino board [7].

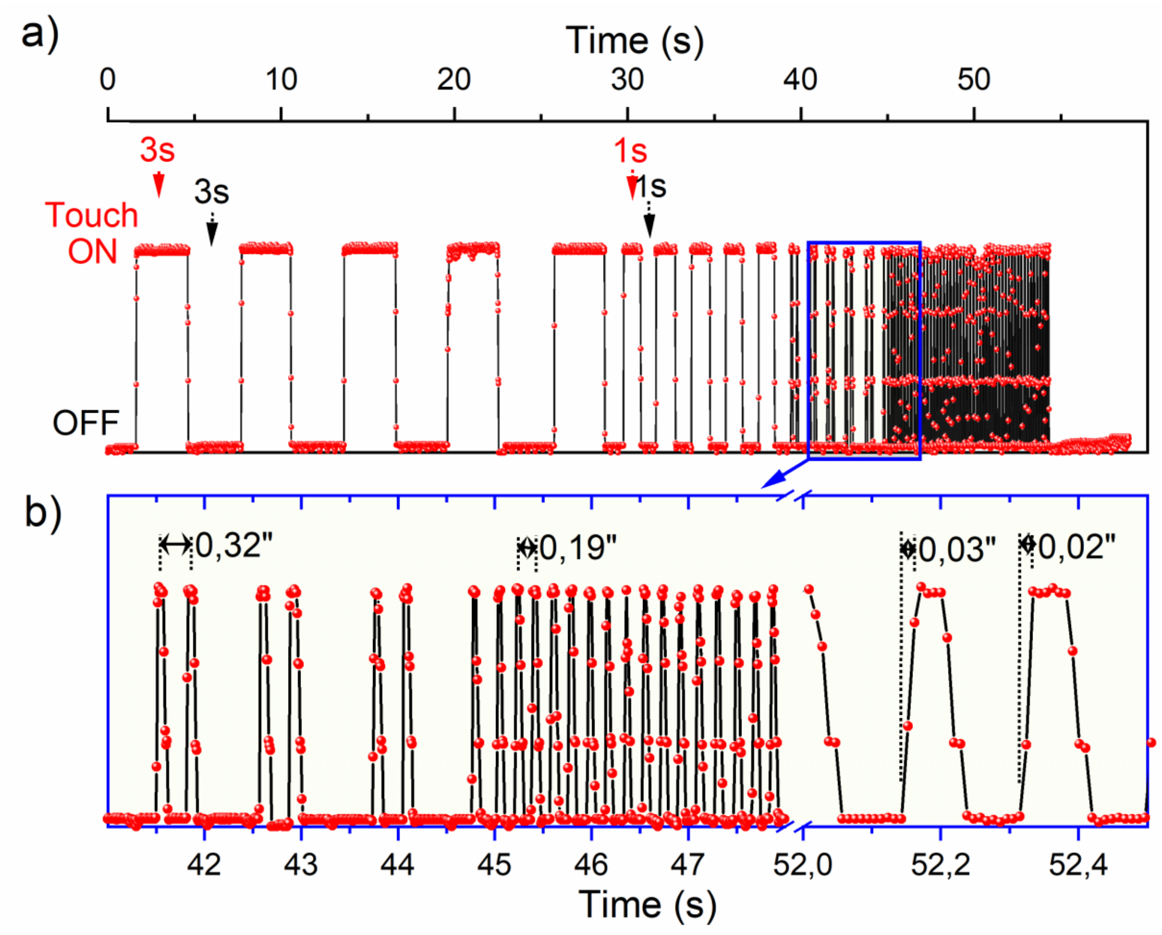

Figure S5. a) touch sensing output from the capacitive sensing device schematized in Figure S4 under ON/OFF touching cycles of different duration (3 s, 1s, and faster cycles); b) time responsivity in dual- and single touch tests.

References.

1. Cesano, F., Groppo E., Bonino F., A. Damin A., Lamberti C., Bordiga S. and Zecchina A. Polyethylene microtubes from silica-fiber-based polyethylene composites synthesized by using an in situ catalytic method, *Adv. Mater.* **2006**, *18*, 3111–3114, doi: 10.1002/adma.200601251.
2. Dhafer, C.E.B., Dhahri M., Mezni A. and Smiri L.S. Surface-enhanced Raman scattering study of PP/Ag nanocomposite developed to prevent postsurgery infection, *J. Raman Spectrosc.* **2018**, *49*, 1445–1451, doi: 10.1002/jrs.5412.
3. Andreassen, E. Infrared and Raman spectroscopy of polypropylene. In *Polypropylene: An A-Z Reference*. J. Karger-Kocsis. Springer: Dordrecht, Poland, 1999 321–328.
4. Jain, S.M., Cesano F., Scarano D. and Edvinsson T. Resonance Raman and IR spectroscopy of Aligned Carbon Nanotube Arrays with Extremely Narrow Diameters Prepared with Molecular Catalysts on Steel Substrates *PCCP* **2017**, *19*, 30667–30674, doi: 10.1039/C7CP06973A.
5. Bokobza, L., Bruneel J.L. and Couzi M. Raman Spectra of Carbon-Based Materials (from Graphite to Carbon Black) and of Some Silicone Composites, *C* **2015**, *1*, 77–94, doi: 10.3390/c1010077.
6. Shimada, T., Sugai T., Fantini C., Souza M., Cançado L.G., Jorio A., Pimenta M.A., Saito R., Grüneis A., Dresselhaus G., *et al.* Origin of the 2450cm−1 Raman bands in HOPG, single-wall and double-wall carbon nanotubes, *Carbon* **2005**, *43*, 1049–1054, doi: 10.1016/j.carbon.2004.11.044.
7. https://www.arduino.cc/. "visited on 10/02/2021"
